# Exenatide: A New Promising Antidiabetic Agent

**DOI:** 10.4103/0250-474X.62228

**Published:** 2010

**Authors:** C. K. Chakraborti

**Affiliations:** Kanak Manjari Institute of Pharmaceutical Sciences, Chhend, Rourkela-769 015, India

**Keywords:** Exenatide, exendin-4, glucagon-like peptide-1, glycosylated hemoglobin, type 2 diabetes mellitus

## Abstract

Exenatide is a unique agent which can effectively control blood glucose levels in type 2 diabetes mellitus without producing dangerous adverse effects. In addition, it can lower body weight which is very essential for the treatment of obese type 2 diabetes mellitus patients. Since it can delay the destruction of islet beta-cells, type 2 diabetes mellitus patients are not rapidly converted to type 1 diabetes mellitus and ultimately appearance of complications of the disease is halted or delayed. Its long-acting-release formula, which would be used once per week, simultaneously retaining all the properties of twice-daily subcutaneous administration, is undergoing clinical trial. This drug is considered as an adjunct to metformin/sulfonylureas/insulin.

Type 2 diabetes mellitus (T2DM) is a progressive metabolic disorder, where, the currently used antidiabetic drugs could not retard the progression of the disease. Therefore, agents, effective in this respect, are needed which can delay such progression. Otherwise, diabetes-associated complications will appear within a short period and there is the possibility of such patients being converted to type 1 diabetes mellitus (T1DM) individuals. Moreover, in spite of multidrug therapy, even with insulin in various combinations and dosage regimens, it has not been possible to achieve proper glycemic control in a significant number of T2DM patients. As the pathogenesis and pathophysiology of the disease is multifactorial, oral antidiabetic agents with different modes of actions have been developed and are in use with various degrees of success. Recently, the role of incretins, particularly that of glucagon-like peptide-1 (GLP-1), in metabolic homeostasis in general and that of glucose in particular, has been firmly established. But, pharmacokinetic studies of endogenous GLP-1 have shown the incretin to have a plasma half-life of only few minutes which makes it unsuitable for routine therapy in T2DM. Hence, attempts were made to find out GLP-1 like substances in other animal species and/or synthesize compounds with GLP-1 like (GLP-1 agonists) action having longer plasma half-life[1].

T2DM, being a disease of overnutrition, its onset and progression are associated intimately with obesity in which there is excess fat accumulation in the abdomen, muscles and liver. Hence, modest weight (wt) loss (approximately 7%) by restricted diet and exercise can check or delay the onset of this disease. It has been observed that in such patients, weight loss decreases fasting and postprandial (pp) plasma glucose levels, glycosylated hemoglobin [HbA1_c_], and need for pharmacotherapy. Unfortunately, most of the currently available antidiabetic drugs, including insulin, cause weight gain. Therefore, pharmacotherapy for obesity, as part of an integrated management plan, is beneficial for maintaining wt loss, optimising glycemic control and probably delaying progression of the disease[2].

Exendin-4 (EX-4), an incretin-mimetic peptide hormone (containing 39 aminoacid residues) having GLP-1 like action[3,4], is secreted in the saliva (oral secretions)[3–6] and concentrated in the tail[3] of Gila monster lizard (*Heloderma suspectum*). This lizard takes food four times a year and during feeding, EX-4, secreted in the saliva, is thought to help its pancreas to switch on[6]. Exenatide (EX) is a synthetic form of EX-4 which shares 53 per cent amino acid sequence similarity with naturally occurring hormone GLP-1[5,7] and acts as a GLP-1 receptor agonist[5].

EX is the first incretin-mimetic compound that has been approved by USA Food and Drug Administration (FDA) in April, 2005, as an adjunctive therapeutic agent to improve glycemic control in T2DM patients who have suboptimal glycemic control with either sulfonylurea or metformin monotherapy[3–5,8] or with sulfonylurea and metformin combination[3].

## Pharmacodynamics of EX:

EX stimulates mammalian receptors for truncated GLP-1 (tGLP-1) with a relatively prolonged action[5] and much longer half-life than GLP-1[4,9]. These two properties made it suitable for treatment of T2DM[4,5,9]. Relatively prolonged action of EX is due to absence of alanine at position 2, as a result of which the compound lacks a recognition sequence for dipeptidyl peptidase-4, the proteolytic metabolizing enzyme, thereby increasing its relative resistance to it[5,7]. GLP-1 receptors are G-protein coupled receptor (GPCR) present on the islet beta-cells. They are also present on tissues other than pancreas like brain, kidney, lungs, heart and major blood vessels. Activation of these receptors on pancreatic beta-cells by GLP-1 leads to augmentation of glucose-induced insulin secretion[10]. Agonist binding signal transduction is effected via stimulation of adenylyl cyclase-cyclic adenosine monophosphate (cAMP) pathway[5,10] leading to activation of cAMP-dependent protein kinase (PKA) and Exchange protein activated by cAMP (Epac) pathways[10].

Couto *et al*[11] have recognized Janus kinase1-Signal transducer and activator of transcription1 (JAK1-STAT1) pathway as novel target of EX-4, where the drug produces downregulation of JAK1-STAT1 transduction mechanism which is an important signaling route mediating the interferon-gamma effects on beta-cell apoptosis in T1DM. These observations indicate that EX-4 treatment may also be beneficial in T1DM, where it may facilitate the protection of beta-cells from cytokine-induced cell death by inhibiting JAK1-STAT1.

**Fig. 1:**
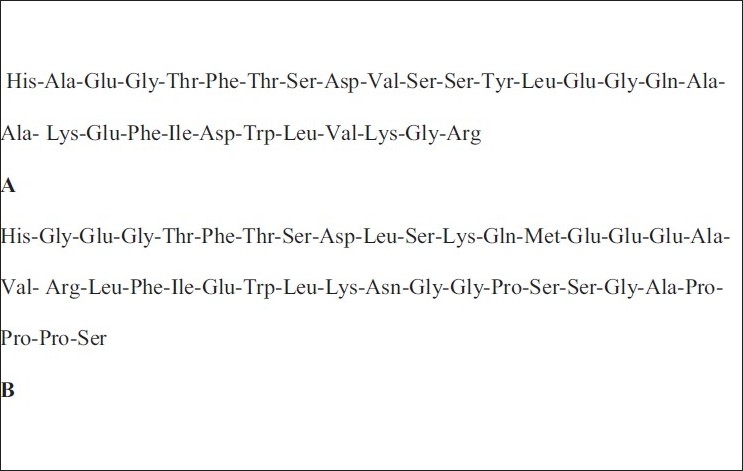
The amino acid sequences of (A) GLP-1 and (B) Exendin-4

The N-terminal region of GLP-1 and EX-4 are almost identical (they share 53 per cent amino acid sequence similarity), a significant difference being in the second amino acid residue alanine in GLP-1 and glycine in EX-4. Another important difference is that EX-4 has an extra nine amino acid residues at its C-terminus[12] as shown in fig. 1.

Some researchers have shown that both of them (GLP-1 and EX) bind and activate the pancreatic GLP-1 receptor (GLP-1R) with similar affinity and potency[13–15]. Continuous GLP-1R activation by EX also enhances insulin synthesis[10]. However, Runge *et al*[15] have shown that the N-terminal extracellular domain of GLP-1R (nGLP-1R) has a ligand binding domain with differential affinity for EX-4 and GLP-1; low attraction for GLP-1 and high affinity for EX-4.

As far as glucose-lowering activity of EX (in animal models) is concerned, it is up to 3000-fold more than exogenous GLP-1[7]. While it may lower blood glucose levels on its own, it can also be combined, as has been mentioned earlier, with other medications to improve plasma glucose control[4].

EX has several mechanisms of action, the ultimate outcome being better glycemic control. Some of the actions are acute (immediate), glucose-dependent insulin secretion, suppression of pp high glucagon concentrations, delayed gastric emptying, inhibition of food intake, and modulation of glucose trafficking in peripheral tissues, while others appear late: weight loss, improved beta-cell mass and function[5,9,16].

EX increases pancreatic response to food leading to enhanced secretion of higher and more appropriate amount of insulin that assists in lowering the pp rise in blood sugar[4]. It controls the blood glucose concentration and can restore glucose-stimulated insulin secretion without excess risk of hypoglycemia[17]. Insulin itself and other antidiabetic drugs which produce their action by increasing the secretion of insulin, can cause dangerous hypoglycemia[4]. But, EX, in spite of its insulin release-stimulating action, does not do so[4,5,18]. Hence, the compound ensures a relatively lesser risk of hypoglycemia, which is an advantage over many antihyperglycemic agents[5,17,19].

EX also checks pancreatic release of glucagon in response to food[4] (without altering the normal insulin/glucagon ratio[20]), which prevents the liver from overproduction of sugar when it is not needed; hyperglycemia does not develop[4]. In this way, hepatic glucose production and subsequent increased insulin requirement are reduced. But, it does not decrease normal glucagon response to hypoglycemia (when needed)[5]. In addition, it delays gastric emptying and thus reduces the rate at which meal-derived glucose appears in the blood stream[4]. It possibly alters hepatic versus peripheral partitioning of glucose metabolism. In this manner it regulates glucose excursion in fed state[5]. The use of EX also leads to the feeling of satiety and fullness, resulting in reduced appetite (prolonged effect), which may manifest as loss of weight[4,5]. Most patients using EX slowly lose weight[4,21], and generally the greatest wt loss is achieved by people who are the most overweight at the beginning of EX therapy[4].

EX decreases pp triglyceride concentrations[5], as well as hepatic fat content both in mice and human being[4]. Fat accumulation in the liver in non-alcoholic fatty liver disease (NAFLD) is related with several metabolic disorders, particularly due to low HDL cholesterol and high triglycerides, as found in T2DM patients[4].

EX helps in differentiation of pancreatic progenitor cells into beta-cells and improves their life span and function by enhancing beta-cell neogenesis and inhibiting their apoptosis[5]. Activation of GLP-1 receptor is accompanied with expansion of beta-cell mass due to stimulation of cell proliferation and promotion of antiapoptotic pathways coupled with beta-cell survival[22]. Enhanced insulin release is speculated to be due in part to a rise in beta-cell mass[8,20]. It is not clear whether the enlarged beta-cell mass results from a decreased beta-cell turnover or increased beta-cell formation or both[8]. EX not only acutely reduces blood glucose but also engages signaling pathways in the islet beta-cells, which cause stimulation of beta-cell replication and inhibition of beta-cell apoptosis[23]. EX has potential capacity to restore the beta-cell mass[24]. When pancreatic islets, prepared from non-diabetic and T2DM subjects, were exposed to EX-4 for 48 h, it was found that the compound had several direct beneficial actions on insulin secretion as well as expression of genes involved in beta-cell function and differentiation[25]. Proliferation and neogenesis of beta-cell cannot be monitored in humans. But, the effect can be inferred from improvements in glucose tolerance and rise of both first and plateau phase of insulin secretory responses in T2DM patients treated with EX-4[10]. Thus, acute effects of EX on the beta-cell responsiveness along with significant decrease in body weight of T2DM patients may have a positive impact on disease progression and potentially lower the risk of associated long-term problems[18].

EX-4 delays progression of diabetes. That is why it has a favourable effect on high blood sugar level which persists for a long time in T2DM patients. It could provide a therapeutic role in diabetic nephropathy that develops due to T2DM[26].

Being encouraged by the effect of EX on beta-cell mass (increased beta-cell proliferation) in T2DM patients, some workers attempted to see the response of it on beta-cell in animals with T1DM. As T1DM is an autoimmune disease, it is associated with near complete beta-cell destruction. At present some evidences suggest that beta-cell regeneration is possible, but ongoing autoimmune damage checks restoration of beta-cell mass. A novel approach to reverse T1DM may be possible by simultaneously blocking autoimmune cytokine damage and supplying a growth-promoting stimulus for beta-cells. For that reason, in one study, researchers combined lisofylline to suppress autoimmunity and EX-4 to increase beta-cell proliferation for treating autoimmune-mediated diabetes in non-obese diabetic (NOD) mouse model. They observed that this therapy effectively checked new-onset diabetes within a week of combined treatment, and even mediated euglycemia up to 145 d after drugs withdrawal. The resultant effect of this therapy was associated with improved beta-cell metabolism and insulin secretion, while decreasing beta-cell apoptosis. It is possible that such unique therapy could become a new strategy to control T1DM in humans[27].

## Pharmacokinetics:

Since EX is a peptide, it needs to be administered parenterally[5]. It is absorbed equally from arm, abdomen or thigh injection sites[8]. The bioavailability of EX after subcutaneous (s.c.) administration has been found to be 65-75 per cent (based on animal studies)[28] (Table 1). After parenteral administration, it reaches a peak concentration in approximately 2 h[8,28] with a duration of action up to 10 h[8]. When a dose of 10 mg is used, a maximum concentration (C_max_) of 211 pg/ml is achieved in 2.1 h (time taken to produce maximum response, i.e., t_max_) along with a mean area under the curve (AUC) of 1036 pgh/ml[5]. After administration of a s.c. dose, the mean apparent volume of distribution is 28.3 l[5,28]. The kidney (by glomerular filtration) is the primary route of elimination followed by degradation of EX by proteolysis[5,28,29]. Its clearance value is 9.1 l/h[5]. Its dosage adjustment is necessary only when creatinine clearance is below 30 ml/min[8] or 1 l/h[5] as found in severe renal failure (end stage renal disease)[5,8]. While the plasma half-life (t_1/2_) of EX is 26 min in humans, it is 1-2 min for biologically active GLP-1. As has been mentioned earlier, this difference is because of penultimate NH_2_-terminal glycine (in EX) instead of alanine (as in GLP-1)[1].

**TABLE 1:** PHARMACOKINETIC DATA OF EXENATIDE

Availability (s.c.) (%)	Vol. dist. (l)	AUC (pgh/ml)	Peak conc. (pg/ml)	Peak time (h)	Clearance (l/h)	Half-life (min)	Duration of action (h)
65-75	28.3	1036	211	2.1	9.1	26	10

## Adverse effects:

Adverse drug reactions of EX are limited and mild to moderate in nature[5,19,30]. These include, nausea, vomiting, diarrhea, jitteriness, dizziness, headache, dyspepsia, uneasiness, decrease in appetite, hypoglycemia (mainly when combined with a sulfonylurea), increased sweating and immunogenic reactions at the injection site[5,28,31]. The chief adverse effects (in approximate percentage of occurrence) are nausea (44%)[8,32], hypoglycemia (20%)[32,33], diarrhea (13%)[32,33] and vomiting (13%)[32,33]. By slowly escalating the dose[5] (or when a target dose of EX is achieved in patients with gradual dose titration[34]), dose-limiting gastrointestinal adverse events like nausea and vomiting can be minimized without loss of glucoregulatory activity.

According to one study report, even doses up to 10 times the recommended dose were accidentally administered in three of the subjects, resulted in severe hypoglycemia requiring parenteral glucose administration. But the recovery in all the cases was uneventful[5].

The use of metformin and EX combination does not increase the incidence of nausea[3] and the occurrence of hypoglycemia was the same as the placebo group[3,34]. On the other hand, mild to moderate hypoglycemia occurs more frequently when EX is combined with a sulfonylurea[3,21,34–36]. Since hypoglycemia occurs in a dose-dependent fashion; patient should be monitored properly for this adverse effect, especially when EX is added to sulfonylurea therapy[32]. It has also been mentioned that the wt loss seen with EX treatment was not due to nausea[3].

Approximately 45 per cent of T2DM patients receiving EX were positive for antiexenatide antibodies, with the majority of them having low titres range (<1/125)[3]. It has also been reported that in most patients antiexenatide antibody concentration is reduced over time[13]. The occurrence of these titres did not seem to have a predictive effect on glycemic response or adverse events[3]. However, due to sequence similarities of EX with endogenous GLP-1 and glucagon (53% and 45% amino acid, respectively), formation of autoimmunity and immunoneutralisation of these related endogenous peptides by antiexenatide antibodies are potential hazards[7]. Moreover, EX may also cause pancreatitis; a warning in this respect has been issued to EX users by USA FDA[6].

## Drug interactions:

As EX delays gastric emptying, caution should be taken when the drug is co-administered with certain drugs like digoxin, lovastatin, lisinopril, acetaminophen, antiinfectives and oral contraceptives[5,28]. It has been suggested to use these agents at least 1 h before the administration of EX with a light meal or snack (if needed)[5].

## Contraindications:

EX is not indicated in T1DM or diabetic ketoacidosis as it is not an insulin substitute. It has not been recommended for diabetics with end stage renal disease (creatinine clearance <30 ml/min) and severe gastrointestinal disorder (like gastroparesis)[3,5].

## Special precautions:

EX is administered in prefilled pen[31]. The pen is required to be refrigerated at a temperature of 2-8° but it is not allowed to be frozen[5]. It should be protected from light[5,31]. Thirty days after the first use, the rest of the drug should be discarded[31].

## Clinical trials:

EX has undergone extensive clinical trials, being used alone or in combination with metformin/sulfonylurea, by several groups of researchers over different time periods with various doses. Such trial results were found to be highly encouraging for therapeutic use of the compound in T2DM either alone or as an adjunct to metformin/sulfonylurea with tolerable adverse effects.

Administering EX once- or twice-daily bolus s.c. injections, Egan *et al*[37] conducted a study taking 10 T2DM patients (insulin naïve), where the drug improved HbA(1)_c_ (P<0.009) after one month of treatment. In another clinical trial lasting 30 w, EX therapy was associated with moderate decrease in mean HbA(1)_c_ level of approximately 0.8 per cent and an average weight loss of about 2 kg compared with baseline[36]. Similarly, Lam and See[32] reported randomized, placebo controlled 30-w clinical studies, where EX promoted glycemic control and improved wt loss of up to 2.8 kg. In some other clinical trials, Jones[38] described statistically significant degree of reduction in HbA(1)_c_ levels (0.3 to 0.7% more than placebo), fasting plasma glucose and body weight (1.4 to 2.3 kg).

Cvetkovic and Polsker[39] have mentioned the results of randomized, controlled, phase 3 trials in T2DM patients. In their study, addition of EX to metformin and/or a sulfonylurea twice-a-day, significantly improved glycemic control and was associated with gradual and important body wt reduction from baseline for up to 2 y. The overall intensity of glycemic control with EX was similar to that of once-a-day insulin glargine or twice-daily biphasic insulin aspart.

Fineman *et al*[40] performed a randomized, multicentric triple blind study enrolling 123 patients who were given EX in gradually increasing doses starting at 0.02 mg/kg thrice-daily, the increment being 0.02 mg/kg per dose every 3 d for 35 d. Use of EX in this manner minimized dose-limiting nausea and vomiting (P<0.001) with no loss of glucoregulatory activity. In another open-label, randomized, controlled trial of 551 patients, EX therapy for 26 w was associated with weight loss of 2.3 kg; however, gastrointestinal symptoms were more frequent in EX group, including nausea (57.1%), vomiting (17.4%) and diarrhea (8.5%). In most of the patients, nausea was mild to moderate and disappeared after a few days or weeks[4]. Incidence of nausea was found to be dose-dependent and was always seen among patients in the clinical trials. It occurred most frequently during 0-8 w and was usually mild to moderate in nature. However, the incidence of severe nausea ranged from 2.7-6.0 per cent. Due to nausea, 1.8-4.0 per cent patients withdrew from the study[3].

In three 30-w double blind, placebo controlled studies, in more than 1400 patients with a mean HbA(1)_c_ of about 8.5 per cent and mean body weight of approximately 99 kg, the use of EX 5 μg and 10 μg s.c. twice-daily with either metformin/sulfonylurea or metformin and sulfonylurea, indicated a decrease in HbA(1)_c_ by 0.6 and 0.9 per cent and body wt by 3.1 and 4.2 kg, respectively as compared to placebo. Long-term extension data in 265 patients showed that the reduction of HbA(1)_c_ was maintained even at 82 w (-1.1% from baseline) and the wt gradually continued to decrease over time (-4.5 kg from baseline). In addition, there were useful effects on lipid profile with small but important reductions in LDL and triglycerides and a rise in HDL cholesterol[3]. Phase 3 clinical trials of EX for 30 w, indicated significantly reduced HbA(1)_c_, fasting and pp plasma glucose compared with baseline when added to metformin/sulfonylureas or a combination of both, with a mean weight loss of about 2 kg. Especially during initiation of therapy with sulfonylureas (not with metformin), hypoglycemia was encountered[34]. In a primary as well as extension study, EX with metformin and/or sulphonylureas, was found to reduce HbA(1)_c_ and body weight which was maintained up to varying periods (from 30 to 82 w). Weight loss was more with EX and metformin than EX and sulphonylureas[41]. Adjunctive EX therapy with metformin/sulfonylurea for ≥ 3 y in T2DM patients achieved sustained improvements in glycemic control, cardiovascular risk factors, and hepatic biomarkers, coupled with progressive wt reduction[42]. In some other clinical trials in T2DM patients who were treated with s.c. EX twice-daily in addition to existing metformin and/or sulfonylurea therapy, it was demonstrated that the drug caused sustained improvement in glycemic control, evidenced by reduction in pp[43] and fasting glycemia[33,38,43], HbA(1)_c_[28,33,38,43] and also modest weight loss[33,38]. As has been mentioned earlier, EX did not increase the likelihood of hypoglycemia when added to metformin therapy[3]. On the other hand, when EX was combined with sulfonylurea, there was a rise in the incidence of hypoglycemic events from 3.3 per cent baseline to 14.4 per cent in patients receiving EX 5 μg twice-a-day and 35.7 per cent in those taking 10 μg EX twice-daily[31].

According to Ratner *et al*[44], EX was well tolerated, producing a long lasting reduction in HbA(1)_c_ and a progressive decrease in weight over 82 w with T2DM failing to achieve glycemic control with metformin alone. In one placebo controlled blind study, Fineman *et al.*[45] took 109 patients with inadequate diabetes control with restricted diet or hypoglycemic agents (metformin or sulfonylureas). They observed HbA(1)_c_ to be less than 7 per cent in 15 per cent of patients as compared to only 4 per cent in the placebo group (P<0.006).

In another study, treatment with EX or insulin glargine for 16 w was accompanied with similar significant improvements in HbA(1)_c_. While EX therapy was associated with notable reductions in body weight and pp glucose excursions, insulin glargine caused significantly greater decrease in fasting serum glucose[46]. Based on the findings of one clinical trial, long-term projections suggested that EX was possibly to be associated with improvement in life-expectancy (of 0.057 y) and quality-adjusted life-expectancy (of 0.442 quality-adjusted life year) compared to insulin glargine. In this trial, EX was associated with a lower cumulative incidence of most cardiovascular disease (CVD) complications and deaths related to CVD than insulin glargine[47].

## Therapeutic uses:

After successful clinical trials, EX has been approved to be used in T2DM patients with either metformin or sulfonylurea or thiazolidinedione monotherapy or with metformin and sulfonylurea combination therapy. Some physicians also administered EX alone or in combination with insulin. During such use, the merits and demerits of the drug has been assessed while administered alone or as an adjunct with others, as mentioned earlier.

In addition to positive therapeutic effects on fasting and pp glucose levels, EX treatment has been found to be associated with significant, dose-dependent decrease in HbA(1)_c_[19] from baseline and gradual reduction in body weight[18,19]. It has been observed that EX-induced glycemic control is achieved in T2DM patients with no/lesser risk of hypoglycemia and weight gain, which are its significant therapeutic advantages[19]. Like Barnett[19], Zinman *et al*[48] also mentioned its similar beneficial effects; moreover, its adverse events were not found to be dangerous. Tsunnekawa *et al*[49] reported that chronic s.c. treatment with EX-4 resulted in significant increase of the insulin contents of the pancreas and the insulin-positive area was retained. From above-mentioned information it is clear that in addition to improvement in glycemic control in T2DM patients, EX can reduce or eliminate the danger of hypoglycemia and weight gain[34]. Since EX therapy often leads to wt loss, this effect further assists in decreasing insulin resistance[50]. It is difficult to get a drug which can lower blood glucose to an appropriate level without inducing a significant associated wt gain and can check the progression of diabetes (earlier it has been mentioned that EX itself can halt progression of the disease). EX and rimonabant are recently developed agents that have both glucose-lowering and body wt reducing properties[51].

EX offers a unique treatment option for T2DM patients who are refractory to metformin or sulfonylurea or both[28,32]. Considering all the actions, adjunctive therapy with EX is a valuable alternative in T2DM patients requiring moderate progress in glycemic control despite treatment with metformin and/or a sulfonylurea. The use of EX with metformin and a sulfonylurea have been found to provide significant improvements in treatment satisfaction and patients' health related quality of life[39]. EX is also considered to be an alternative therapy for those patients who cannot tolerate other antidiabetic drugs[32]. In two open-label, randomized, multicentric comparative (insulin) controlled trials in T2DM patients suboptimally controlled with metformin and a sulfonylurea, treatment with EX (5 μg twice-daily for 4 w and 10 μg thereafter) and an insulin analogue (glargine or biphasic insulin aspart) resulted in similar effects in HbA(1)_c_. On the other hand, EX produced decrease in body weight while insulin analogue caused weight gain. So, EX is a treatment option in insulin-naïve patients with T2DM and who are overweight and suboptimally controlled by metformin and sulfonylurea[52].

Another useful finding is that EX effectively treats obese subjects with T2DM on insulin, leading to weight loss and decrease in levels of HbA(1)_c_, systolic blood pressure, triglycerides and high-sensitivity C-reactive protein (CRP)[53]. Chronic EX treatment has been found to increase insulin sensitivity and protection against high-fat-induced insulin resistance[54]. Administration of insulin (daily) is inconvenient; hypoglycemia and wt gain are its known adverse effects. Moreover, it has a possible role in atherogenesis[38]. On the other hand, EX administration is comparatively easier and incidence of hypoglycemia is low. Instead of weight gain, the drug actually reduces it. This is an important advantage, since most of the T2DM patients are already obese or overweight[55]. Virji[56] has also mentioned greater wt loss in obese patients with T2DM who are receiving EX compared with those taking sulfonylureas, thiazolidinediones or insulin. Instead of causing atherogenesis, EX exerts a favourable effect on lipid profile[3]. Comparing insulin glargine with EX in some T2DM patients, important findings have been obtained. The average HbA(1)_c_ concentrations fell just above 7 per cent in both groups (1.1% drop from baseline). But the body weight in patients using insulin glargine increased by a mean of 1.8 kg during the study, while that of subjects using EX declined by an average of 2.3 kg. Such investigation results suggest that insulin glargine and EX produce similar benefits as far as HbA(1)_c_ levels are concerned, but EX has added advantage of weight loss. During this study it was also observed that nausea occurred in about 9 per cent of patients using insulin glargine, while it was found in 57 per cent of patients receiving EX. The incidence of vomiting was about 4 per cent of T2DM in insulin glargine group and 17 per cent in patients using EX (P<0.001). Comparable hypoglycemic rate was seen in both the groups, 6.3 events per patient-year in insulin glargine group and 7.3 cases per patient-year in the group using EX. However, the incidence of nocturnal hypoglycemia was slightly more in the patients taking insulin glargine[57]. In another comparative study, it has been reported that EX therapy was associated with significant decrease in both body wt and pp glucose excursions, whereas insulin glargine was linked with a significantly more reduction in fasting serum glucose. These observations present additional information to take treatment decisions in T2DM patients who are potential candidates for either therapy[46].

Similar to biphasic insulin aspart, EX treatment also resulted in HbA(1)_c_ decrease. Since EX therapy provided better glycemic control, it is considered to be a potential alternative for the treatment of T2DM. Moreover, its therapeutic advantage is wt reduction[58]. EX can also be administered in combination with thiazolidinediones and may be considered as an alternative to insulin in patients requiring additional therapy[34]. Although EX is an adjunctive therapy for T2DM, preliminary evidence indicates that its glucoregulatory effects may be similar in the absence of oral antidiabetic therapy. An important observation is that even EX twice-daily monotherapy resulted in glycemic improvements and reductions in body wt comparable to that of EX combination use with metformin in T2DM patients. EX 10 μg twice-daily for 28 d caused significant mean decrease in HbA(1)_c_[30].

In USA, one long-term cost-effectiveness of Ex was estimated in T2DM patients, where it was found to improve cost-effectiveness. In addition to sustained reduction in HbA(1)_c_, the extra clinical effects of improved lipid levels, systolic blood pressure, and lowered body mass index all positively contributed to the cost-effectiveness of EX[59].

## Dose:

The initial recommended dose of EX (in combination with metformin or sulfonylureas) is 5 μg (s.c.) twice-a-day, administered within 60 min before the morning and evening meals[3,5,28,31]. The drug should not be injected postprandially[3]. After 1 mo, the dose may be increased to 10 μg (s.c.) twice-daily[3,31] which produces better diabetes management[60].

When EX is used with metformin in T2DM patients, metformin dose may not be adjusted. On the other hand, when it is administered with sulfonylurea, dose reduction of sulfonylurea should be considered because such combination is liable to cause more hypoglycemia[31]. EX decreases insulin requirement in some patients and may delay the need to resume insulin in others[61]. As has been mentioned earlier, EX is easier to administer than insulin because of its prefilled pen design and simple dosing schedule[56].

## Long-acting EX:

One of the important drawbacks of EX therapy is its twice-daily parenteral administration. To overcome this disadvantage a long-acting injectable preparation of the compound has been formulated which can be used once-a-week[3,62], even once-a-month[5]. Long-acting release preparation of EX (EX LAR) is currently in the phase of clinical development. Several comparative clinical trial results have documented the similar beneficial results of this form of EX administered subcutaneously once-a-week/once in two weeks/once-a-month with that of EX, which is used twice-daily (BID).

Phase 3 clinical trials have been conducted to find out the plasma concentration and effects of EX LAR preparations administered at different time intervals. In these dosage forms, EX is being placed inside a biodegradable polymeric microsphere, which produces a sustained-release formulation that can be injected once-a-week, once-every-other week, and once-a-month. Such preparations have been found to maintain adequate plasma concentrations of the active drug for weeks to months after a single dose. Following injection of EX LAR, a small amount of active drug diffuses out of this polymeric microsphere due to hydration of the polymer, and there is continued stable release of active drug due to progressive breakdown of the microsphere over time[63].

In a 16 w study, 2 doses of EX LAR administered once-weekly were well tolerated and simultaneously achieved dose-dependent improvements in HbA(1)_c_ and body weight (loss of weight)[3]. In T2DM subjects, EX LAR formulation administered once-a-week, provided adequate 24-h glycemic control and wt reduction with only mild nausea (as found with EX BID). None of the EX LAR treated patients withdrew from the study[62].

Kim *et al*[62] used two doses (0.8 mg and 2.0 mg) of EX LAR for their study. They found that treatment with 2 mg EX LAR (but not with 0.8 mg) reduced body weight. It indicates that higher EX concentrations are necessary for its effect on weight. The magnitude of pp glucose excursions reduced as much as four fold with 2 mg EX LAR (compared with placebo LAR). HbA(1)_c_ levels were decreased with 2 mg dose.

EX LAR once-weekly therapy is promising in T2DM, because the preparation retains all the benefits of EX BID intact without being administered frequently[62]. No other antidiabetic medication comes closer to lowering HbA(1)_c_ as effectively as EX LAR along with the wt loss profile, this may improve further with longer-term use. Considering patient's compliance, once-weekly formulation of the drug (EX LAR) may replace EX BID for the treatment of T2DM patients. Whether these useful effects of EX are maintained in the longer-term use and it has effects on pancreatic beta-cell regeneration in humans remain to be studied. So, longer-term large scale studies are needed to gain further insight into treatment with EX LAR.

Another option to bypass twice-daily use of EX is its intranasal administration. It requires an aqueous mixture of EX and a delivery enhancer selected from the group consisting of a solubilizer, a chelator and a surfactant[64]. As a surfactant at least one alkyl glycoside and/or at least one saccharide alkyl ester may be used. When it is admixed with a drug, the surfactant stabilizes the biological activity and enhances bioavailability of the drug[65]. These novel, highly effective and non-irritating alkyl saccharide transmucosal delivery enhancing agents, like Intravail, have overcome the two primary limitations of intranasal delivery like mucosal irritation and poor bioavailability. Such formulations offer the promise of more convenient, more effective and safer therapeutics for patients and physicians alike[66]. Very recently, Gedulin *et al*[67] have reported other promising routes of administration like sublingual and inhalation.

## CONCLUSIONS

EX is a synthetic GLP-1 receptor agonist. When administered subcutaneously, it was found to increase glucose-dependent insulin secretion, suppression of pp high glucagon concentration, delayed gastric emptying, inhibition of food intake, modulation of glucose trafficking in peripheral tissues and wt loss with mild to moderate hypoglycemia and gastrointestinal adverse effects.

From the clinical trial results provided by different research groups and therapeutic responses observed by different physicians, EX appears to be a useful agent in the management of T2DM. Clinical trials on T2DM patients have been conducted both for short (about 30 d)[37,40] as well as long (26 w or more)[3,4,32,34,39,41,42,44] periods, employing the compound (at different doses) alone or in combination with metformin/sulfonylureas. In all these trials EX, administered subcutaneously, has been found to possess significant beneficial effects on important antidiabetic parameters like reduction of HbA(1)_c_[36–38], improved glycemic control including both fasting and pp plasma glucose level[33,34,38] and weight loss[32,36,38], along with mild to moderate persistent hypoglycemia[31,34], nausea and vomiting[3,4,40] which disappeared in due course or persist, though mildly. Combination therapy produced better results[3,33,34] in comparison to monotherapy either with EX[37,40]/metformin[41]/sulfonylurea[41,68], in addition to improved lipid profile[3]. Some workers even showed that the addition of EX is effective in T2DM patients when metformin[44,45]/sulfonylurea[45] alone failed. Ray *et al*[47] compared the long-term benefits of EX with that of insulin glargine and found the compound to be more effective than insulin glargine with respect to improvements in life-expectancy, quality-adjusted life-expectancy and lower cumulative incidence of CVD complications and CVD-related deaths (probably because of its ability to improve lipid profile).

After the FDA approval for marketing, EX was used for treatment of T2DM by many physicians either alone or in combination with metformin/sulfonylureas/thiazolidinediones/insulin, but its utility as a first line drug is yet to be defined[20]. When used alone, the drug was found to reduce HbA(1)_c_, fasting as well as pp blood glucose and body wt along with less hypoglycemia, nausea and vomiting (gastrointestinal symptoms)[19]. Moreover, it was found to increase the insulin contents of pancreas along with retention of insulin-positive area[49]. When EX was added to metformin/sulfonylureas/both in T2DM patients, better response was observed in relation to reduction of HbA(1)_c_, plasma glucose (both fasting and pp) and wt[33,39]. Moreover, improvement in health-related quality of life was observed[39,53]. Hence, the drug can be considered as a good adjunct to metformin/sulfonylureas in T2DM patients. Several results of EX with insulin therapy in obese T2DM patients have been documented where the combination was found to reduce HbA(1)_c_, body weight, systolic blood pressure, triglycerides and high-sensitivity CRP[53] along with increased insulin sensitivity and protection against high-fat-induced insulin resistance[54]. Though insulin is the best agent for controlling blood sugar level in diabetic patients, EX has been shown to possess some advantages over insulin glargine and biphasic insulin aspart in T2DM subjects. While reducing HbA(1)_c_ and providing significant glycemic control like insulin, EX, in contrast to insulin, has been found to reduce wt[3,46,55–58], improve lipid profile[3] and cause less hypoglycemia[46]. In addition, EX administration is comparatively easier than insulin (though both of them used subcutaneously) because of the improved device for its s.c. administration and simple dosing schedule[56]. Minshall *et al*[59] found EX to produce sustained decrease in HbA(1)_c_ in addition to improved lipid profile, decreased systolic blood pressure and body mass index; all of which positively contributed to the cost-effectiveness of the drug.

From the above observations it can be concluded that EX is an ideal adjunct to metformin/sulfonylureas/insulin in T2DM patients where it can potentiate the antidiabetic action without increasing hypoglycemia with the additional benefit of weight loss, thereby decreasing insulin resistance which is a major problem in obese T2DM patients. During such adjunctive therapy, though the patient requires additional daily s.c. injections and suffers from gastrointestinal adverse effects (mild nausea and vomiting)[36,69], associated weight loss[36,69,70] and positive cost-effectiveness[59] satisfies the patients. Major drawback of EX is its twice-daily s.c. administration when this treatment schedule is followed. But this problem has been solved by formulation of a long-acting form of EX (EX LAR) which can be used once-a-week[62]; even once-a-month[5] without compromising the beneficial effects of EX BID.

One unique and exceptional property of EX has been demonstrated by several study groups, where the drug has been found to halt the progression of degeneration[26] (apoptosis[23,27]) of insulin secreting islet beta-cells as well as stimulates their regeneration[23], both on T2DM[23,26] and T1DM[27]. Convincing study reports in this respect are awaited. If proved, this action of EX would eliminate or reduce the major progressive pathology of diabetes mellitus, thereby bringing great relief to many diabetes mellitus patients as well as physicians, who are facing a tremendous challenge daily to treat the most complicated disease, diabetes mellitus, both type 1 and 2.
